# Letter to the editor regarding “Giant mucinous cystadenocarcinoma of ovary in a young woman: a case report and review of literature” Volume 14 2024

**DOI:** 10.3389/fonc.2024.1443334

**Published:** 2024-08-23

**Authors:** Lisa Feinstein

**Affiliations:** Camelot Animal Hospital, Davie, FL, United States

**Keywords:** ovarian cancer, ovarian mucinous adenocarcinoma, ovarian borderline malignancy, ovarian cystadenoma, ovarian cystadenocarcinoma, MAC

Our 19-year-old daughter Kate died on 23 May 2024 of stage 4 primary ovarian mucinous cystadenocarcinoma 1 year after diagnosis. She first felt abdominal pain while on vacation in Maine in August 2021. CT scan and ultrasound showed a large fluid filled cyst on her right ovary (without concerning solid parts or septation). On her 17th birthday, she had a laparoscopic cystectomy for an 18-cm right ovarian cyst. The pathology for this was (and verified later) mucinous cystadenoma (August 2021). The cyst recurred and was confirmed on ultrasound in late 2022, and she had a laparoscopic right ovarian cystectomy for another 18-cm cyst in mid-March 2023. The pathology report at this time revealed predominantly mucinous cystadenoma, but there was focal borderline tumor and a small amount of mucinous cystadenocarcinoma. By May 2023, when the gynecological oncologist staged her, mucinous carcinoma was in her peritoneal washing and infracolic omentum, making her stage 3. Her cancer antigens were normal until November 2023, when she developed massive ascites and her CA125 went just above 50, soon after finishing six rounds of FOLFOX chemotherapy. When she was administered hyperthermic intraperitoneal chemotherapy cytoreduction at MD Anderson in December 2023, mucinous carcinoma was noted throughout her abdomen/intestinal tract, giving her a Peritoneal Cancer Index score of 39. How does a gynecologist suspect mucinous cancer and know to remove the entire adnexa in a very young woman with normal CA125 and benign imaging? Mucinous cystadenocarcinoma is rare and does not always elevate cancer antigens, as in my daughter’s case. My daughter may have been saved if her entire ovary was removed without spillage at the first or second surgery. However, most gynecologists I spoke to would have just done a cystectomy the first and even the second time as she was an asymptomatic, healthy 18-year old, and her cyst was read as benign at the first surgery. You have one chance with primary ovarian mucinous cancer and that is removing the adnexa without spillage at the first surgery, but, unfortunately, my daughter Kate did not have that. If primary ovarian mucinous cancer is diagnosed beyond stage 1, the prognosis is grim. Removing an ovary unnecessarily is a small sacrifice I would gladly have made if I knew cancer was a possibility and we may have been saving her life. How can gynecologists prevent this tragedy in other young women?

